# Complexes of Ag and ZnO nanoparticles with BBR for enhancement of gastrointestinal antibacterial activity through the impacts of size and composition

**DOI:** 10.1039/d3ra00053b

**Published:** 2023-02-20

**Authors:** Hue Thi Nguyen, Tuyet Nhung Pham, Le Thi Le, Tien Khi Nguyen, Anh-Tuan Le, Tran Quang Huy, Thuy Thi Thu Nguyen

**Affiliations:** a Phenikaa University Nano Institute (PHENA), Phenikaa University Hanoi 12116 Vietnam huy.tranquang@phenikaa-uni.edu.vn thuy.nguyenthithu@phenikaa-uni.edu.vn +84 978960658 +84 924926886

## Abstract

This study introduces the bioformulations of Ag/BBR and ZnO/BBR complexes against pathogenic bacteria in the gastrointestinal tract. Without the use of toxic reduction agents, Ag and ZnO NPs were prepared using an electrochemical method and then facially mixed with BBR solution to form Ag/BBR and ZnO/BBR complexes. BBR molecules are strongly conjugated with Ag and ZnO NPs through coordinated bonding and electrostatic interaction. As a result, the presence of BBR significantly influenced the nanoparticle growth, resulting in the formation of core/shell structured Ag/BBR and ZnO/BBR NPs with small particle sizes. The antibacterial test showed that BBR, Ag, or ZnO components all contributed to the increase of antibacterial ability of Ag/BBR and ZnO/BBR NPs against both methicillin-resistant *Staphylococcus aureus* (MRSA) and *Salmonella enteritidis* (*S. enteritidis*). The bactericidal ability of Ag/BBR and ZnO/BBR complexes against MRSA was exhibited even at a concentration of four-fold dilution (corresponding to 1.25 g L^−1^ of BBR and 46.25 mg L^−1^ of Ag) and two-fold dilution (corresponding to 2.5 g L^−1^ of BBR and 10 mg L^−1^ of ZnO), respectively, while that of the Ag/BBR complex against *S. enteritidis* showed at a concentration of two-fold dilution corresponding to 2.5 g L^−1^ of BBR and 92.5 mg L^−1^ of Ag. The results obtained in this study support that Ag/BBR and ZnO/BBR complexes can be potential therapeutic agents against gastrointestinal infections.

## Abstract 2

The presence of BBR resulted in the formation of small core/shell structured Ag/BBR and ZnO/BBR nanoparticles and improvement of antibacterial activity.

## Graphical abstract

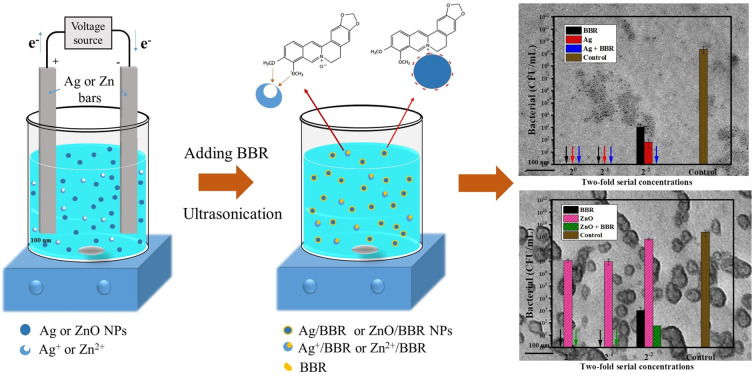

## 1. Introduction

Gastroenteritis is an illness caused by the inflammation of the stomach and intestines, typically because of bacterial or viral infection. The major symptoms of gastroenteritis are stomach pain, diarrhea, and vomiting. The use of medicinal plants to treat various gastroenteritis problems has been recorded in the history of many countries in the world. The most famous are Chinese and Indian herbs. Medicinal plants contain various types of phytochemicals, such as alkaloids, quinones, terpenoids, glucosinolates, oligosaccharides, and phenolic compounds. Many of them could decrease intestinal permeability, which is one of the major causes of intestinal diseases, including Crohn's disease and ulcerative colitis.^1,2^ Besides, they could modulate oxidative stress and enzymatic activity within the gastrointestinal tract as well as reduce pro-inflammatory cytokine production.^1,3,4^ Berberine (BBR) is an isoquinoline alkaloid, and a well-known traditional medicine to treat diarrhea and gastroenteritis.^5^ The anti-diarrheal action of BBR was attributed to its antitoxin and antibacterial activity against pathogenic bacteria in gastrointestinal such as *Vibrio cholera*, *Escherichia coli* (*E. coli*), *Staphylococcus aureus* (*S. aureus*), *Pseudomonas aeruginosa*, *Streptococcus faecalis*, *Shigella flexneri*, *Shigella sonnei*, *Shigella dysenteriae*, *Shigella boydii*, and *Clostridium difficile*.^5–9^ In addition, one of the mechanisms explaining the anti-inflammatory action of BBR is its inhibition effects on the growth of Gram-negative intestinal bacteria, such as *E. coli*, *Klebsiella pneumonia*, and *Proteus mirabilis*.^5,10^ In the role of a protective agent in digestive diseases, BBR could preserve the intestinal epithelial barrier from the inflammatory response.^11^ In addition, BBR has been effective in the treatment of other diseases, including cancer, type 2 diabetes, hypercholesterolemia, cardiovascular, and neurological diseases.^11,12^ However, the crucial drawbacks of BBR are its poor solubility, leading to its low bioavailability and high-dose drug requirement, in turn highly causing side effects.

Recently, nanomaterials have emerged as potential drug-resistance antimicrobial agents because their nanoscale size could improve or generate unique chemical, electrical, thermal, mechanical, or optical characteristics. Interestingly, the high specific surface area and the functionalized surface of nanomaterials could enhance their chemical reactivity and mobility, increasing drug-target delivery for therapy.^13^ BBR nanoparticles showed a significant increase in solubility, dissolution rate, and antibacterial activities against Gram-positive and Gram-negative bacteria, and yeasts in comparison with raw BBR.^14^ Meanwhile, metal nanoparticles including silver, gold, copper, zinc, and their oxides have shown a broad-spectrum antibacterial activity because their nano-size can easily penetrate the biofilm, direct contact with the bacterial cells, and release the loaded antimicrobial agents causing the death of the surviving cells.^15^ Silver nanoparticles (Ag NPs) are one of the most intense metal nanomaterials for antimicrobial treatment. Ag NPs can be prepared by eco-friendly techniques using plant extracts, and biological or microbial agents instead of highly toxic reducing chemicals to meet the strict requirements in bio-applications.^16^ Zinc oxide nanoparticles (ZnO NPs) are recognized as a bio-safe nanomaterial applied in biology and medicine with some interesting possibilities such as antitumor, antimicrobial, antifungal and anti-inflammatory activities, wound healing ability, antidiabetic properties, *etc.*^17,18^ It was reported that the minimum inhibitory concentration of biosynthesized Ag and ZnO NPs against various bacterial strains varied in the ranges of 31.25 ÷ 62.5 mg L^−1^ and 440 ÷ 854 mg L^−1^, respectively.^19,20^ Although the antibacterial activity of ZnO NPs is not superior to that of other metal materials, the worthy property of ZnO NPs is their good biocompatibility with human cells.^21^ Variations in structure, size, and composition of nanomaterials greatly improve their performance in different applications,^22,23^ as well as modulate their impact on health and the environment.^24^ Besides, there are several methods to increase the antimicrobial action of nanoparticles have been proposed, including a combination of different antibacterial compounds; physic-chemical modification of nanoparticle surface; change in the size or shape of nanoparticles. The presence of Ag NPs in the BBR/Ag NPs/silk fibroin layer used for coating calcium phosphate scaffolds could significantly increase the antibacterial effect of scaffolds with suitable biocompatibility and osteoinductive activities for bone tissue engineering.^25^ Recently, bioformulations of BBR combined with Ag NPs or ZnO NPs studied mainly for their possibility in cancer therapy,^26–28^ against hepatic and renal damage during the diabetic condition,^29^ and anti-Covid-19.^2^ For cancer treatment based on a molecular-based targeting approach, Bhanumathi *et al.*^26^ formulated folic acid- and BBR-loaded Ag NPs nanomaterial, in which BBR was a cancer therapeutic agent, Ag NPs was a drug-nanocarrier, and folic acid was used to modulate the release of BBR molecules into the specific cancer site. As the result, the nanomaterial showed a significant restraint of tumor progression *in vivo*. The ZnO NPs/BBR complex showed anti-Covid-19 by inhibiting the virus entry, replication, and assembly. Furthermore, it could prevent respiratory co-bacterial infection in Covid-19 patients.^2^

This study aims to green synthesis of Ag/BBR and ZnO/BBR complexes by a facile procedure: Ag and ZnO colloids were prepared by electrochemical method and then conjugated with BBR molecules to form Ag/BBR and ZnO/BBR complexes. The intermolecular interaction in Ag/BBR and ZnO/BBR complexes was investigated by several analysis methods, including XRD, Raman, UV-Vis, TEM, and cyclic voltammetry. The antibacterial activity of Ag/BBR and ZnO/BBR complexes against pathogenic bacteria in the gastrointestinal tract, namely, methicillin-resistant *Staphylococcus aureus* (MRSA, Gram-positive bacteria) and *Salmonella enteritidis* (*S. enteritidis*, Gram-negative bacteria) was examined. In this study, we also explained the enhanced antibacterial effect of Ag/BBR and ZnO/BBR complexes against both Gram-positive (Gram +) and Gram-negative (Gram −) bacteria based on the interaction between each component in the complexes and the interaction between complexes and bacterial cells. Interestingly, BBR helped to increase the antibacterial activity of Ag and ZnO complexes against Gram-positive bacteria. Meanwhile, Ag and ZnO complexes led to ameliorative effects of BBR against Gram-negative bacteria. Our results suggest that the synthesized Ag/BBR and ZnO/BBR complexes are potential therapeutic agents against gastrointestinal tract infections.

## 2. Materials and methods

### 2.1 Materials

BBR chloride (pharmaceutical primary standard) and trisodium citrate (Na_3_C_6_H_5_O_7_, purity > 99%) were purchased from Sigma Aldrich. Glycerol was supplied by Fisher Scientific (purity > 99%). Nutrient broth and nutrient agar were provided by Titan Biotech, India. Bi-distilled water through a Milli-Q® system was used.

Two bulk silver bars (99.999% purity) and two bulk zinc bars with dimensions (*L* × *W* × *T*) of 150 × 10 × 0.5 mm were used as electrodes for the electrochemical process.

Two bacterial strains, namely, MRSA (Gram-positive bacteria) and *S. enteritidis* (Gram-negative bacteria) were provided by the Department of Bacteriology, National Institute of Hygiene and Epidemiology, Vietnam.

### 2.2 Methods

#### 2.2.1 Preparation of colloidal Ag NPs and ZnO NPs by an electrochemical method

Colloidal Ag NPs were prepared by an electrochemical method described in our previous publication.^30^ Briefly, two silver bars were immersed in 0.02 wt% Na_3_C_6_H_5_O_7_ solution in a 250 mL glass beaker. Two silver bars played a role as two electrodes were placed apart at a distance of 5 cm. The electrochemical process was performed at a direct current voltage source of 9 V for 2 h at room temperature. Subsequently, the obtained solution was centrifuged at the speed of 5000 rpm for 5 min. The supernatant liquid in the centrifuge tubes was collected and used for the preparation of Ag/BBR NPs. Colloidal ZnO NPs were prepared according to a similar process to forming colloidal Ag NPs. By atomic absorption spectroscopy analysis, the Ag and Zn concentration of the prepared Ag and ZnO NPs colloids were about 185 and 20 mg L^−1^, respectively.

#### 2.2.2 Preparation of BBR, Ag/BBR and ZnO/BBR complexes

**Fig. 1:**
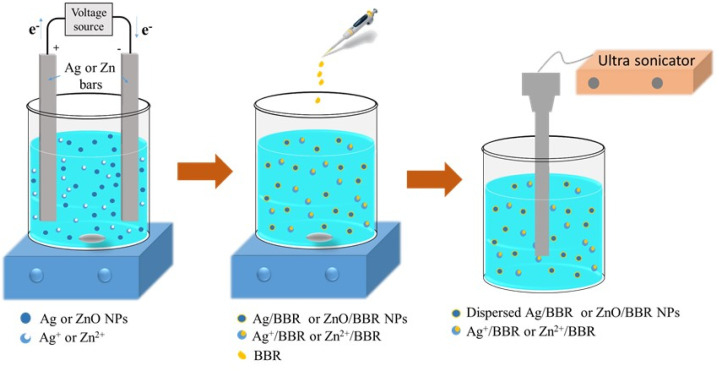
A new experimental design for the fabrication of core/shell Ag/BBR and ZnO/BBR NPs.

BBR NPs were formed by an antisolvent precipitation process, which was in detail described in our previous study.^31^ Ag/BBR and ZnO/BBR NPs were prepared by a similar process as follows: BBR solution was prepared by dissolving 100 mg of BBR in 1 mL of glycerol at 37 °C. Then, this solution was slowly dropped into beakers containing 19 mL of fresh colloidal Ag NPs or ZnO NPs under a stirring condition of 1000 rpm. After that, these solutions were sonicated by a probe ultrasound sonicator at conditions of 50% ultrasound amplitude for 15 min under external ice cooling (UWave-2000, Sineo). The schematic diagram of the preparation of core/shell Ag/BBR and ZnO/BBR complexes is presented in Fig. 1.

#### 2.2.3 Characterization of BBR, Ag/BBR and ZnO/BBR complexes

The UV-Vis absorption spectra of BBR NPs, Ag NPs, Ag/BBR complex, ZnO NPs, and ZnO/BBR complex were measured at the wavelength from 200 to 550 nm by a UV-Vis spectrophotometer (6850 UV/Vis, Jenway).

The chemical interaction between colloidal Ag NPs, ZnO NPs and BBR was investigated using a Raman spectrometer (MacoRAM, Horiba) in the wavelength range of 500 – 3000 cm^−1^.

The morphology of Ag NPs, Ag/BBR NPs, ZnO NPs, and ZnO/BBR NPs was characterized by transmission electron microscopy (TEM, JEM1010, JEOL).

For X-ray diffraction (XRD) measurement, the samples were centrifugated at 10 000 rpm for 10 minutes to obtain concentrated solutions. These solutions were dropped onto 15 × 15 mm glass slides and dried at 40 °C. Then, the samples were analysed by an XRD unit (EQUINOX 5000 – Thermo Scientific) at a scan range of 5° < 2*θ* < 80°.

Cyclic voltammetry (CV) measurement was performed on the Palmsens 4 electrochemical workstation in an ambient atmosphere. Briefly, Palmsens screen printed electrodes (SPE) were separately dipped into the solutions of aqueous phosphate buffer (PBS 0.1 M), Ag colloids, ZnO colloids, Ag/BBR complex and ZnO/BBR complex and then applying a potential range of −0.3 and 0.6 V at a scan rate of 50 mV s^−1^.

#### 2.2.4 Assessment of antibacterial activity of colloidal Ag NPs, ZnO NPs, Ag/BBR complex, and ZnO/BBR complex

The antibacterial activity of BBR NPs, Ag NPs, Ag/BBR complex, ZnO NPs, and ZnO/BBR complex was tested against two bacterial strains causing gastroenteritis, namely, MRSA (Gram + bacteria) and *S. enteritidis* (Gram − bacteria), at a concentration of 10^5^ colony-forming units (CFU) per mL, respectively.

**Fig. 2:**
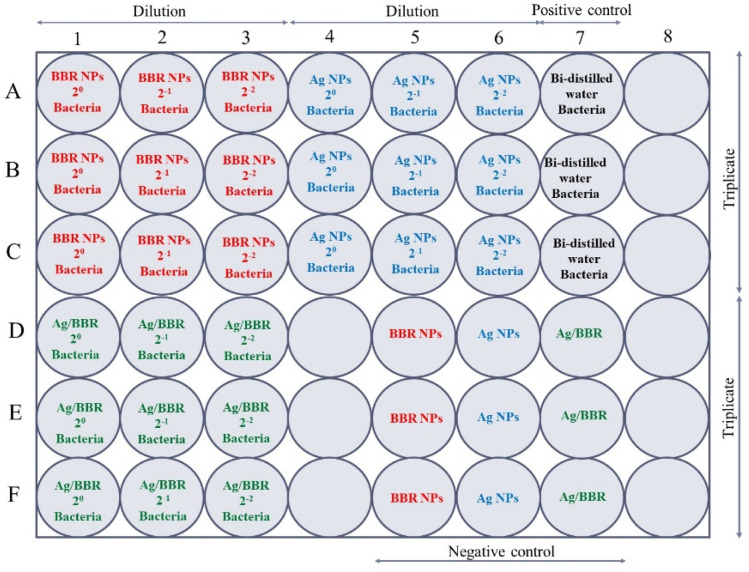
The experimental design for the antibacterial test of BBR NPs, Ag NPs, and Ag/BBR complex at three different concentrations against MRSA and *S. enteritidis* bacterium.

The antibacterial test of BBR NPs, Ag NPs, and Ag/BBR complex at three different concentrations (two-fold serial concentrations) against each bacterial strain was performed in a sterilized 48-well plate designed as follows (Fig. 2): all wells of the 48-well plate were added 180 μL of the nutrient broth. In row A, the first three wells were filled with 200 μL of BBR NPs with different concentrations (marked as BBR NPs 2^0^, BBR NPs 2^−1^, and BBR NPs 2^−2^). Similarly, 200 μL of Ag NPs with different concentrations (marked as Ag NPs 2^0^, Ag NPs 2^−1^, Ag NPs 2^−2^) were added into three next wells. In this row, the well in column 7 served as a positive control when it was filled with 200 μL of bi-distilled water. Then, 20 μL of a bacterial strain was added to these wells. In row B and row C, the above experiment steps were repeated for triplicate measurements. In row D, 200 μL of Ag/BBR complex with different concentrations (marked as Ag/BBR 2^0^, Ag/BBR 2^−1^, Ag/BBR 2^−2^) and 20 μL of a bacterial strain was added into the first three wells. The wells in columns 5, 6, and 7 contained BBR NPs, Ag NPs, and Ag/BBR complex served as negative controls. Triplicate measurements were performed by repeating all samples of row D in rows E and F. Then, the plate was incubated at 37 °C for 24 h. A similar experiment was used for testing the antibacterial activity of ZnO NPs, and ZnO/BBR complex at different concentrations.

The antibacterial effectiveness of BBR NPs, Ag NPs, Ag/BBR complex, ZnO NPs, and ZnO/BBR complex was compared based on the number of viable bacteria after being treated by these samples. The viable bacteria were evaluated by counting the bacterial colonies growing on the agar surface. Briefly, after incubating at 37 °C for 24 h, the solutions in the 48-well plate were diluted many times in the physiological saline. 100 μL of the diluted solutions were spread onto the agar surface in Petri discs and incubated at 37 °C. After 24 h, the number of bacteria colonies was counted and from that, the viable bacterial concentration in inoculated solutions was calculated.

#### 2.2.5 Bacterial cell observation by TEM

The ultrastructural characteristics of two bacterial strains (MRSA and *S. enteritidis*) treated with Ag NPs, Ag/BBR complex, ZnO NPs, and ZnO/BBR complex were observed by the following process: 10^5^ CFU mL^−1^ of bacterial strains were inoculated on nutrient agar for 24 h at 37 °C. Then, 1 mL of Ag NPs, Ag/BBR complex, ZnO NPs, and ZnO/BBR complex solutions were spread on the colonies of bacteria and kept for 1 h at room temperature. 2.5% glutaraldehyde/cacodylate was used to fix these bacterial colonies for 1 h at room temperature. Ultrathin 70 nm-thick sections of colonies were obtained using an ultramicrotome (Ultracut UC6, Leica) and observed using TEM.

#### 2.2.6 Statistical analysis

The data obtained from the antibacterial test was subjected to a one-way analysis of variance (ANOVA) for the statistical significance of 3 groups (*p* < 0.05). Error bars represent standard deviations.

## 3. Result and discussion

### 3.1 Characterization of BBR, Ag/BBR, and ZnO/BBR complexes

**Fig. 3:**
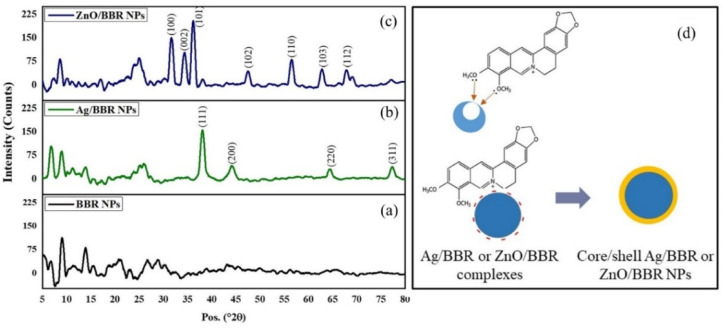
XRD pattern of (a) BBR NPs, (b) Ag/BBR complex, (c) ZnO/BBR complex, and (d) the proposed structure of Ag/BBR and ZnO/BBR complexes.

The X-ray diffraction (XRD) pattern of BBR, Ag/BBR, and ZnO/BBR complexes were analyzed to confirm the crystallinity and phase formation (Fig. 3).

The XRD pattern of Ag/BBR complex (Fig. 3b) shows diffraction peaks at scattering angles (2*θ*) of 38.01, 44.23, 64.57, and 77.47° corresponding to 111, 200, 220, and 311 planes, respectively, of the face-centred cubic crystal structure of Ag (JCPDS card no. 04-0783). In Fig. 1c, diffraction peaks characterized to 100, 002, 101, 102, 110, 103, and 112 planes of the hexagonal wurtzite structure of ZnO were found at 31.69, 34.44, 36.19, 47.62, 56.59, 62.89, and 67.99°, respectively (JCPDS card no. 36-1451). In the XRD pattern of BBR complexes (Fig. 1a), the characteristic peaks of BBR NPs appeared at 2*θ* of 6.79, 9.13, and 13.90 which indicates the crystalline phase of BBR. However, the intensity of these peaks in the XRD pattern of Ag/BBR and ZnO/BBR complexes was lower, showing the semi-crystalline form of BBR in Ag/BBR and ZnO/BBR complexes. This result suggests that the presence of Ag^+^ and Zn^2+^ or their reduced forms (Ag and ZnO) affected the recrystallization of BBR in an aqueous solution. During the electrochemical process of two Ag bars, Ag^+^ was released by the oxidative dissolution of the sacrificial Ag anode and then migrated to the cathode to the reductive formation of silver atoms (Ag^0^). The Ag NPs were formed *via* nucleation of Ag^0^ and subsequent growth because of attractive van der Waals forces between Ag atoms. Similarly, during the electrochemical process of two Zn bars, Zn^2+^ was formed and immediately reacted with oxygen to produce ZnO NPs. As the result, two solutions were obtained right after an electrochemical process of Ag and Zn bars composed of minor residual Ag^+^, Ag NPs and minor residual Zn^2+^, ZnO NPs, respectively. When adding BBR into these solutions, their components, namely Ag^+^, Ag, Zn^2+^, and ZnO could form complexes with BBR through charge transfer or coordinately bonding. The proposed structure of these complexes is displayed in Fig. 3d. Specifically, Ag^+^ or Zn^2+^ could bind strongly to BBR molecules because of the charge transfer from the methoxyisoquinoline group as donor moiety to the Ag^+^ or Zn^2+^ as acceptor moiety.^32^ Meanwhile, the methoxy O atom of BBR could donate free electrons to the Zn atom to form a coordinate covalent bond.^2,28^ Besides both Ag and ZnO NPs possessed negative charge because of the attachment of citrate molecules on the surface,^30^ which caused an electrostatic interaction between negatively charged Ag and ZnO NPs and the positive-charged quaternary ammonium group of BBR. As the result, the presence of Ag^+^, Ag, Zn^2+^, or ZnO in the solution led to reducing the crystallinity of BBR. The sharp and intense peaks characterized by Ag and ZnO were clearly observed, indicating the good formation of their crystalline phase.

**Fig. 4:**
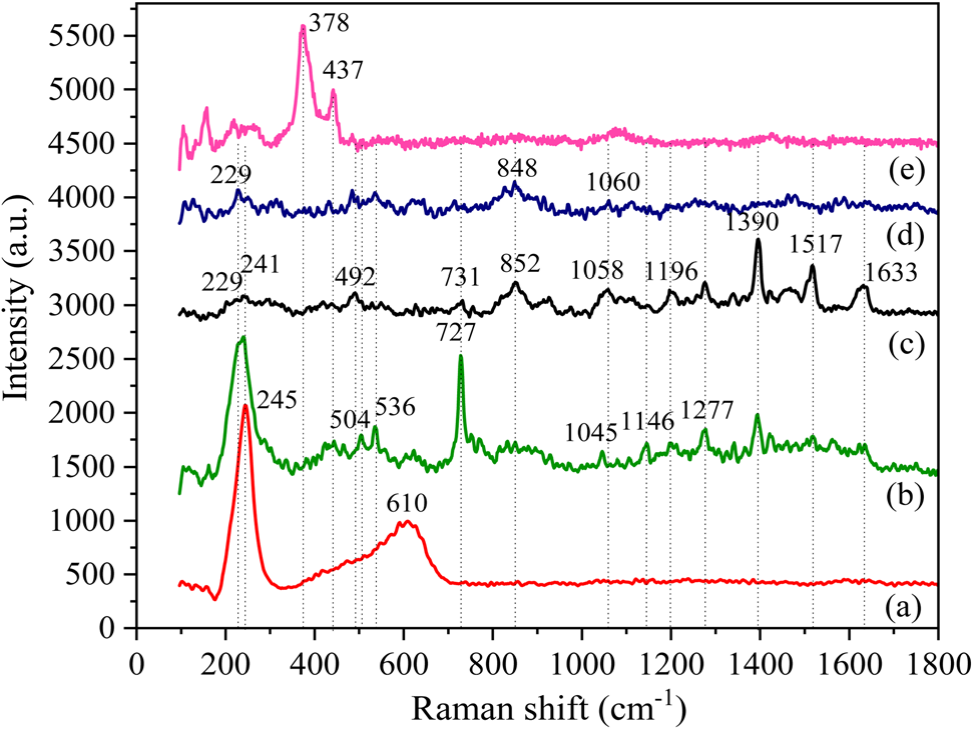
Raman spectra of (a) Ag NPs, (b) Ag/BBR complex, (c) BBR NPs, (d) ZnO/BBR complex, and (e) ZnO NPs.

To further verify the intermolecular interaction in Ag/BBR and ZnO/BBR complexes, the Raman analysis of BBR, Ag, ZnO, Ag/BBR complex, and ZnO/BBR complex was performed (Fig. 4). The Raman spectrum of BBR NPs and their peak assignments are shown in Fig. 4 c and Table 1, respectively. Interestingly, the characteristic peaks of BBR had a significant change in intensity and location when BBR combined with Ag and ZnO.^33,34^ In the case of Ag/BBR complex, there was a disappearance of peaks at 492 and 1058 cm^−1^ characterized by BBR, while the intensity of peaks located at 852, 1390, 1517, and 1633 cm^−1^ drastically reduced. Moreover, some new peaks clearly appeared at 536 and 1045 cm^−1^, accompanied by the enhancement in the intensity of peaks centred at 540, 727, 1146, and 1277 cm^−1^. This evidence presents the strong interactions between BBR molecules and Ag^+^ or Ag NPs, which is in agreement with the above XRD analysis. The vibration peak of BBR at 731 cm^−1^ was notably increased in intensity along with shifting towards a lower wavelength (727 cm^−1^) due to the effect of the surface plasmon absorption of Ag NPs, which is responsible for the enhancement in the Raman signals of BBR attached on the surface of Ag NPs.^35^ It is reported that the entire molecular plane of BBR can adsorb on the surface of Ag colloid^34,35^ and then the Raman emission from BBR is enhanced because of the large local electromagnetic field caused by the surface plasmon excitation of Ag NPs. Besides, the charge-transfer resonance of BBR–Ag complex at Fermi energy under the laser excitation can theoretically account for Raman signal enhancement.^35^ In the case of ZnO prepared by electrochemical process, two detectable peaks located at 378 and 437 cm^−1^ were dominantly assigned to the A_1_(TO) and E^high^_2_ modes of ZnO wurtzite crystal, respectively.^36^ These peaks did not appear in the Raman spectrum of ZnO/BBR complex, possibly due to the loss of translational symmetry caused by intrinsic defects of ZnO NPs complexed with BBR.^37,38^

**Table 1:** The assignment of vibration peaks in Raman spectrum of BBR NPs^a^

Chemical structure	Wavenumbers (cm^−1^)	Peak assignment
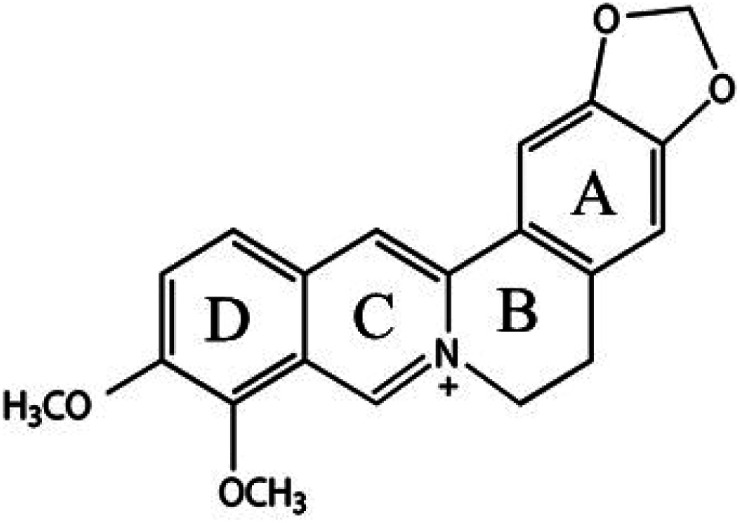	731	Out-plan *δ* of rings B and C
	852	Out-plan *δ*[C, H] of ring B
	1058	*δ*[C–C–C] of ring E, *ν*[C–C] of ring C, *ν*[C–O] of methoxy groups
	1196	*ν*[C–N] of ring D, *ω*[CH_2_] of ring C
	1277	*ν*[C–N] of ring C, *ρ*[–OCH_3_]
	1390	*ν*[C–C], *ν*[C–N] of ring C, *ρ*[CH_3_, CH_2_], *ν*[C–O] of methoxy groups
	1517	*ν*[C=C] of ring E, *ν*[C–N] of ring C
	1633	*ν*[C=C] of ring D, *ς*[–OCH_3_]

a Abbreviation of vibrations: *δ* – bending, *ν* – stretching, *ρ* – rocking, *ω* – wagging, *ς* – scissoring.

**Fig. 5:**
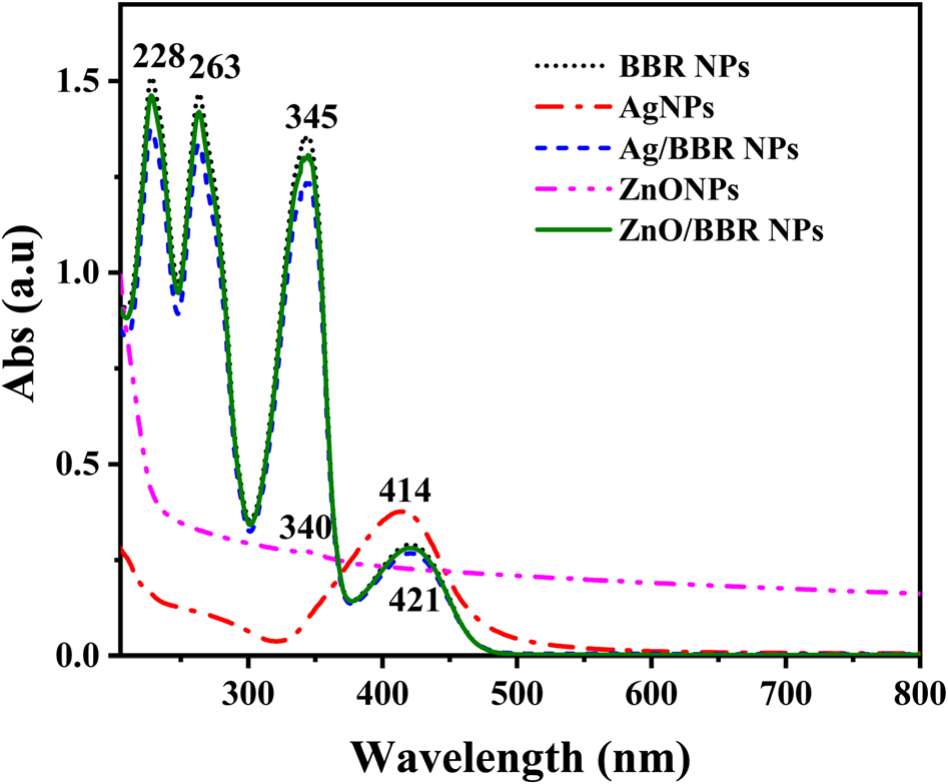
UV-visible absorption spectra of Ag, ZnO, BBR, Ag/BBR complex, and ZnO/BBR complex in aqueous suspension.

To confirm the formation of Ag and ZnO NPs prepared by the electrochemical method as well as investigate the interaction between BBR molecules with Ag and ZnO NPs, UV-visible absorption spectra of Ag, ZnO, BBR, Ag/BBR complex, and ZnO/BBR complex in aqueous suspension were measured and the results are shown in Fig. 5. Benefiting from the surface plasmonic effect of nanoparticles, the absorption peak of Ag and ZnO NPs were identified at 414 and 340 nm, respectively. While the characteristic absorption peaks for BBR centered at 228, 263, 345, and 421 nm, in which three first bands were attributed to π–π* transition of isoquinoline moiety and the latter band correspond to the n–π* transition in BBR molecule.^39,40^ In the case of Ag/BBR and ZnO/BBR complexes, their absorption spectra were similar to that of BBR NPs, but had slightly lower intensity. The characteristic absorption peaks of Ag and ZnO NPs were not found in the absorption spectrum of Ag/BBR and ZnO/BBR complexes. This can be explained by the adsorption of BBR molecules on the surface of Ag and ZnO NPs to form Ag/BBR and ZnO/BBR core/shell structured nanoparticles, resulting that the characteristic absorption peaks of Ag and ZnO NPs overlapped by the strong absorption peaks of BBR.

**Fig. 6:**
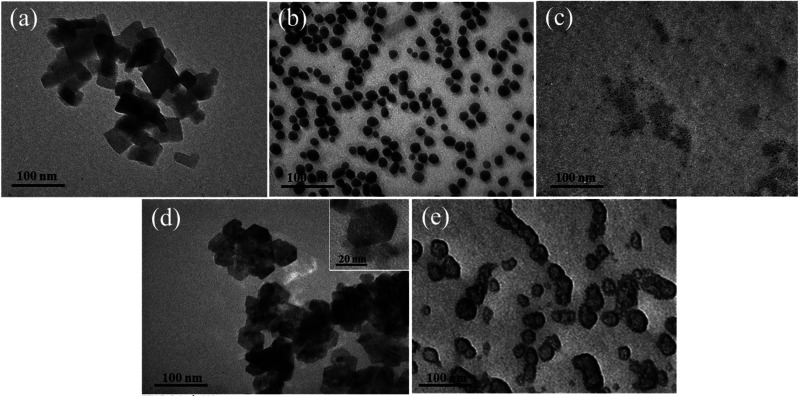
TEM images of (a) BBR NPs, (b) Ag NPs, (c) Ag/BBR NPs, (d) ZnO NPs, and (e) ZnO/BBR NPs.

**Fig. 7:**
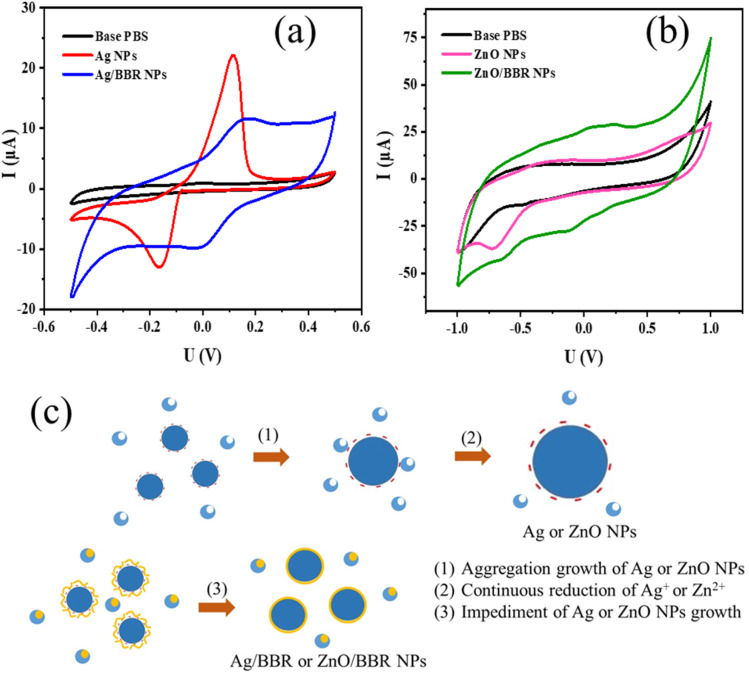
Cyclic voltammetry of (a) Ag NPs and Ag/BBR complex, (b) ZnO NPs and ZnO/BBR complex, and (c) the proposed mechanism for the formation of Ag NPs and core/shell Ag/BBR NPs.

TEM images of Ag, ZnO, Ag/BBR NPs, and ZnO/BBR NPs were recorded to evaluate their size, shape, and surface morphology (Fig. 6). It can be observed that the BBR NPs had a rectangular shape with two dimension lengths lower than 100 nm. Synthesized by the electrochemical method, the Ag NPs exhibited a spherical shape with particle size between 10 and 30 nm, while the ZnO NPs appeared in regular hexagon shape with a size ranging from 20 to 40 nm. Interestingly, adding BBR into freshly prepared Ag NPs or ZnO NPs solutions led to a significant change in the morphology and dispersity of Ag NPs or ZnO NPs. As shown in Fig. 6c, the particle size of individual Ag NPs was notably reduced to several nanometers and the Ag NPs tended to clump together in clusters. The prepared ZnO NPs were also smaller in size and changed from a hexagon shape to a spherical shape. The ZnO NPs seem to be covered by a thick and rough layer, which was illustrated by a darker region of ZnO NPs as seen in Fig. 6e. This outer layer could be formed by the adsorption of BBR on the surface of ZnO NPs. The change in shape and size of Ag and ZnO nanoparticles may be due to the presence of BBR affected on the subsequent growth state of nanoparticles in solutions obtained after the electrochemical process. This assertion is confirmed by the analytical results of cyclic voltammetry in Fig. 7. Cyclic voltammetry describes the electron transfer processes of inorganic complexes during electrochemical reduction and oxidation, that is, it is specific to the redox behaviour of coordination compounds. The voltammograms of Ag NPs showed an anodic peak at 0.12 V corresponding to the oxidation of Ag to Ag^+^ deposited on the electrode and a cathodic peak at −0.16 V, corresponding to the reduction of Ag^+^ into Ag. Apparently, the anodic peak was markedly higher than the cathodic one, indicating the higher concentration of formed Ag in comparison with that of residual Ag^+^ in solution after the electrochemical process. By this time, the Ag NPs gradually grow to a bigger size due to the reduction of residual Ag^+^ on the surface of the growing Ag NPs through a heterogeneous catalytic mechanism.^41^ However, the presence of BBR in the solution caused the shift of the anodic peak toward higher potential (approximately +0.18 V) along with the significant decrease of peak current, suggesting the higher barrier to electrotransfer between the electrode and Ag due to the BBR layer surrounding the Ag NPs, causing by strong complexing of Ag by BBR molecules. In addition, the voltammogram of Ag/BBR complex recorded a broad cathodic peak with a relatively low current density, indicating a deceleration in the reduction process. In other words, BBR retarded the reduction of Ag^+^ and therefore inhibition the subsequent size growth of Ag NPs formed in the electrochemical process. A similar phenomenon was observed in the voltammogram of ZnO NPs and ZnO/BBR complex (Fig. 7b). In the case of ZnO NPs, there was only a cathodic peak appeared at −0.76 V, corresponding to the reduction of ZnO *via* the following reaction:ZnO + H_2_O + 2e → Zn + 2OH^−^

It is worth noting that the current of the reduction peak of ZnO significantly decreased in the presence of BBR, which was caused by the formation of BBR adsorption layer on the surface of ZnO NPs, leading to an impediment to the transport of electrons between electrode and ZnO NPs and preventing the additional growth in the size of formed ZnO NPs. In the voltammogram of ZnO/BBR complex, new cathodic and anodic peaks can be observed at potentials of −0.12 V and +0.24 V, respectively. It is proposed that the appearance of these peaks due to the redox cycle of berberine occurred on the surface of ZnO NPs. In other words, ZnO NPs were considered as a catalyst for the oxidation of BBR in an aqueous medium, in which ZnO NPs acted as an electron mediator leading to accelerating electron transfer between BBR molecules and electrodes.^42–44^ Maikap *et al.*^42^ also reported that a reduction peak raising in the cyclic voltammetry was the result of the oxidation of catechol on ZnO surface.

The proposed mechanism for the formation of Ag NPs prepared by electrochemical process and the effect of BBR on the size of Ag NPs is shown in Fig. 7c. As mentioned above, BBR could form a complex with Ag^+^ or Zn^2+^, leading to retard the reduction of Ag^+^ or Zn^2+^, which is necessary for the growth of Ag NPs or ZnO NPs, respectively, by heterogeneous catalytic mechanism. Furthermore, the formation of the adsorption layer of BBR on the surface of newly formed Ag NPs or ZnO NPs could interfere with their continuous growth to a bigger size caused by the aggregation mechanism. Both of these effects resulted in the formation of Ag NPs or ZnO NPs with small sizes. A similar mechanism was described in some scientific reports dealing with the synthesis of small-size Ag NPs or ZnO NPs by the reaction systems including polymeric agents or plant extracts.^41,45,46^

The formation of the strong complex between BBR and Ag or Ag^+^, Zn^2+^, ZnO caused the loss of BBR's nanoparticle morphology. BBR molecules could not crystallize into BBR nanocrystals but instead formed a layer of BBR crystals adsorbed on the surface of the Ag or ZnO NPs.

### 3.2 Antibacterial assay

**Fig. 8:**
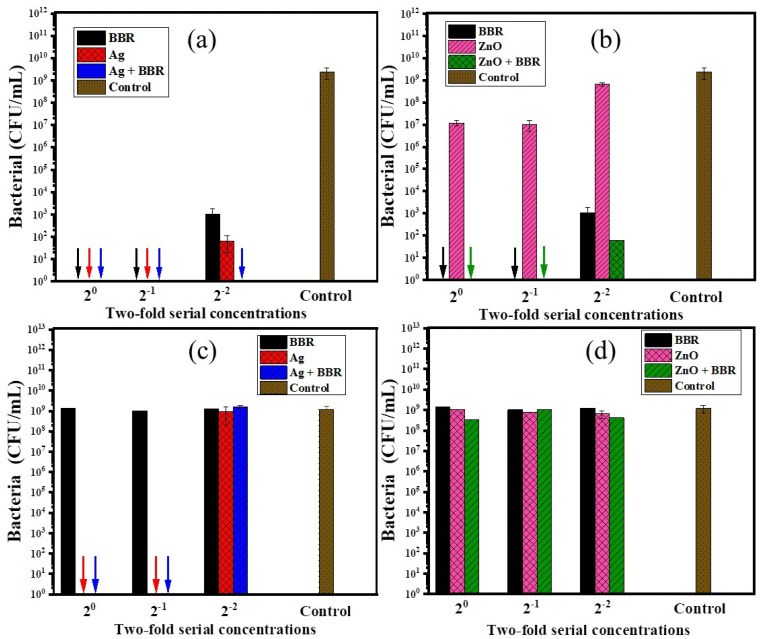
The antibacterial effectiveness of (a) BBR, Ag, and Ag/BBR complex against *S. aureus*, (b) BBR, ZnO, and ZnO/BBR complex against *S. aureus*, (c) BBR, Ag, and Ag/BBR complex against *S. enteritidis*, and (d) BBR, ZnO, and ZnO/BBR complex against *S. enteritidis* at different concentrations.

BBR, Ag, and ZnO NPs have been shown to have antibacterial activity against a wide range of Gram − and Gram + bacteria. In this study, the antibacterial activity of Ag and ZnO NPs prepared by the electrochemical method and their complexes with BBR was evaluated, thereby clarifying the impact of each component in the complexes on their antibacterial ability. The antibacterial effectiveness of BBR, Ag, ZnO, Ag/BBR complex, and ZnO/BBR complex at different concentrations against two strains of bacteria, namely MRSA (Gram +) and *S. enteritidis* (Gram −), was shown in Fig. 8. The result indicates that Ag and BBR NPs alone exhibited bactericidal activity against MRSA at their stock and two-fold diluted concentrations. At four-fold dilution, Ag NPs were able to inhibit the growth of MRSA more than BBR NPs, meanwhile Ag/BBR complex could kill all of MRSA at the same concentration. Although ZnO NPs showed considerably lower antibacterial activity against MRSA than Ag NPs, the antibacterial activity of ZnO/BBR complex still followed a similar trend to that of Ag/BBR complex; that is superior to the antibacterial activity of either BBR NPs or ZnO NPs at four-fold dilution. The possible explanation for this result is the influence of the smaller particle size and the surface modification of the Ag and ZnO NPs by active BBR molecules. Another possible reason for the increased antibacterial activity of Ag/BBR complex is the greater residue of Ag ions which have stronger antibacterial activity than its nanoparticle form.^47^ Against *S. enteritidis*, BBR NPs did not show antibacterial ability, even at the highest concentration of BBR NPs, while ZnO NPs had an inhibitory effect and Ag NPs showed bactericidal activity at a bacterial concentration of 10^5^ CFU mL^−1^. Therefore, the result indicates that the antibacterial effect of Ag/BBR and ZnO/BBR complexes against S. enteritidis was mainly contributed by Ag and ZnO components. Ag and ZnO nanoparticles simultaneously played two crucial roles: they acted as carriers of BBR drug and served as effective antibacterial agents. The slight effect of ZnO/BBR particle size on antibacterial efficacy against *S. enteritidis* was modestly shown at the stock and two-fold dilution concentrations.

In summary, the surface modification of Ag and ZnO NPs by BBR layer fairly enhanced the antibacterial ability against MRSA because BBR molecules had a better affinity for the cell membrane of Gram + bacteria. Meanwhile, thanks to Ag and ZnO components, the antibacterial effect of the Ag/BBR and ZnO/BBR complexes against Gram − bacteria was exhibited. The minimum bactericidal concentration of Ag/BBR could be reached at low concentration (four-fold dilution corresponding to the BBR and Ag concentrations of 1.25 g L^−1^ and 46.25 mg L^−1^, respectively), while that of ZnO/BBR was obtained at higher concentration (two-fold dilution corresponding to the BBR and Zn concentrations of 2.5 g L^−1^ and 10 mg L^−1^, respectively).

**Fig. 9:**
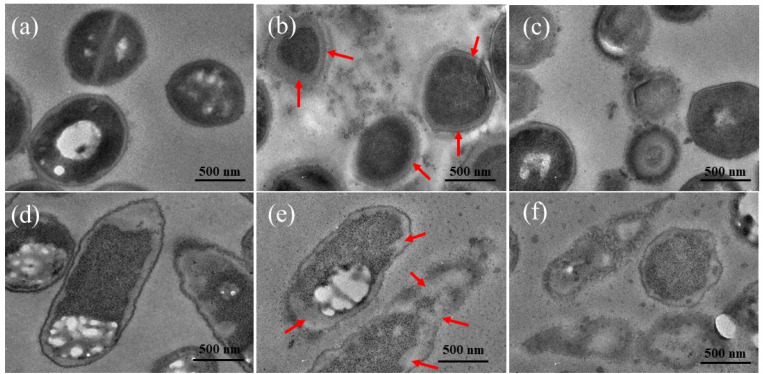
Cross-sectional TEM images of MRSA cells (a) untreated, (b) treated with Ag/BBR complex, (c) treated with ZnO/BBR complex, and *S. enteritidis* cells (d) untreated, (e) treated with Ag/BBR complex, (f) treated with ZnO/BBR complex.

Morphological and ultrastructural changes of bacterial cells untreated and treated by Ag/BBR, and ZnO/BBR complexes were examined by TEM observation of the ultrathin section (Fig. 9). The untreated MRSA and *S. enteritidis* had normal shapes and clearly identified margins of the cell envelope, periplasmic region and cytoplasmic membrane. The MRSA cells treated with Ag/BBR complex present septal peptidoglycan thickening accompanied by the lack of the septal central dense layer (arrows on Fig. 9b). This phenomenon has been reported most frequently in *S. aureus*.^48^ Meanwhile, treated *S. enteritidis* formed a large distance between the cytoplasmic membrane and outer membrane (arrows on Fig. 9e). This result has been found on the Gram-cell envelope treated by some antibacterial agents.^48^ Intracellular vacuoles identified by round-shaped electron-transparent areas that appeared in the cytoplasm of bacterial cells treated with ZnO/BBR complex were observed in Fig. 9c and f. Besides, other ultrastructural changes were seen including cell wall damage and leakage of cytoplasm, resulting in cell collapse following treatment with Ag/BBR and ZnO/BBR NPs. Particularly, it can be seen that Ag/BBR and ZnO/BBR complexes are more attached around MRSA cells than *S. enteritidis* cells. This is due to the difference in the composition of the bacteria cell wall; that is the cell wall of MRSA contains teichoic acids, whose backbone has negatively charged phosphate groups. Therefore, the highly negatively charged MRSA cell wall gave more electrostatic interaction with the positively charged ammonium groups of BBR molecules adsorbed on the surface of Ag and ZnO NPs. As a result, the antibacterial activity of Ag/BBR and ZnO/BBR complexes against MRSA was higher than against *S. enteritidis* cells.

## 4. Conclusions

Ag/BBR and ZnO/BBR complexes were synthesized by electrochemical method with the addition of BBR. BBR molecules formed complexes with Ag and ZnO NPs through coordinated bonding and electrostatic interaction, forming the outer shell of these particles. It suggested that the formation of the adsorption layer of BBR on the surface of newly formed Ag NPs and ZnO NPs could interfere with their continuous growth to a bigger size. As a result, Ag/BBR and ZnO/BBR complexes showed increased antibacterial activity against MRSA because they had small particle sizes with the outer BBR layer having a better affinity for Gram + bacteria. The bactericidal ability of Ag/BBR and ZnO/BBR complexes against MRSA was exhibited even at a concentration of four-fold and two-fold dilution, respectively. For Gram − bacteria, Ag played the crucial role as the antibacterial component of Ag/BBR complex, killing all *S. enteritidis* bacteria at a concentration of two-fold dilution. In the other words, BBR, Ag, or ZnO components all contributed to the synergic antibacterial ability against both Gram + and Gram − bacteria. Therefore, Ag/BBR and ZnO/BBR complexes can be considered potential therapeutic agents against gastrointestinal infections. The effect of particle size and BBR shell layer on the toxicity of the Ag/BBR and ZnO/BBR nanoparticles will be further evaluated *in vitro* before moving on to the tests *in vivo* and *ex vivo*.

## Author contributions

Thuy Thi Thu Nguyen and Tran Quang Huy designed, coordinated this research and drafted the manuscript. Hue Thi Nguyen, Tuyet Nhung Pham, Le Thi Le, and Khi Tien Nguyen carried out experiments and data analysis. Anh-Tuan Le revised the manuscript. The authors read and approved the final manuscript.

## Conflicts of interest

The authors have no competing interests or personal relationships that could have appeared to influence the work reported in this paper.

## Supplementary Material
